# Gut Microbiome in Alzheimer’s Disease: from Mice to Humans

**DOI:** 10.2174/1570159X22666240308090741

**Published:** 2024-03-22

**Authors:** Chang Liang, Resel Pereira, Yan Zhang, Olga L. Rojas

**Affiliations:** 1 Department of Gastroenterology, West China Hospital of Sichuan University, Chengdu, China;; 2 Department of Immunology, University of Toronto, Toronto, ON, Canada;; 3 Krembil Research Institute, University Health Network, Toronto, ON, Canada

**Keywords:** Alzheimer’s disease (AD), gut microbiome, diet, fecal microbiota transplantation (FMT), prebiotics, probiotics

## Abstract

Alzheimer's disease (AD) is the most prevalent type of dementia, but its etiopathogenesis is not yet fully understood. Recent preclinical studies and clinical evidence indicate that changes in the gut microbiome could potentially play a role in the accumulation of amyloid beta. However, the relationship between gut dysbiosis and AD is still elusive. In this review, the potential impact of the gut microbiome on AD development and progression is discussed. Pre-clinical and clinical literature exploring changes in gut microbiome composition is assessed, which can contribute to AD pathology including increased amyloid beta deposition and cognitive impairment. The gut-brain axis and the potential involvement of metabolites produced by the gut microbiome in AD are also highlighted. Furthermore, the potential of antibiotics, prebiotics, probiotics, fecal microbiota transplantation, and dietary interventions as complementary therapies for the management of AD is summarized. This review provides valuable insights into potential therapeutic strategies to modulate the gut microbiome in AD.

## 1 INTRODUCTION

The gastrointestinal tract is the largest organ involved in absorption of nutrients, drugs, and protection against ingested pathogens. In the intestine, the gut microbiome (GM) contributes to the complex ecosystem with over 1000 bacterial species, archaea, fungi, and protozoans which are known to play an important role in health and disease.

Growing research sequencing technology such as 16S rRNA analysis and metagenomics over the last 20 years, have expanded the overall knowledge around GM composition in health and disease. 16S rRNA sequencing allows bacterial community profiling (phyla level) but results can be biased due to differences in experimental design, sample collection, type of DNA extraction, sequencing and analysis [1], explaining sometimes variable reports in the literature. Recently, the shotgun metagenomics has appeared as a key technique to identify GM at species/strain level and to provide information about the biological functions of each bacterial community present in the sample [2]. Several large-scale projects such as the NIH-Human Microbiome Project have shed some light on the human microbiome [3], revealing that the most abundant bacteria phyla in the human gut are *Bacteroidetes* and *Firmicutes* [4]. Significant changes in the abundance and distribution of the healthy bacteria phyla may contribute to human diseases such as, Type 2 Diabetes mellitus [5], Central nervous system (CNS)-related disorders [6] and inflammatory bowel disease [7]. The gut microbiome that mainly drives an overall inflammatory state in the organs is known as “proinflammatory GM” and it is highly linked with diseases whereas the most prevalent bacteria in healthy people that brings a homeostatic intestinal environment are known as an “anti-inflammatory GM”. Current interest in the gut-brain axis and causality between intestinal microbiome and diseases in the central nervous system, has given rise to controversial questions about the importance of GM in Alzheimer’s Disease (AD). In this review, it is aimed to summarize available pre-clinical and clinical literature exploring changes in GM composition and its impact on future therapeutics for AD.

## 2 PRE-CLINICAL MODELS OF ALZHEIMER'S DISEASE: CHANGES IN GUT-MICROBIOME COMPOSITION AND RELATED EFFECTS

Gut dysbiosis, which refers to the loss of non-pathogenic/ healthy GM and the rise of pathogenic/unhealthy GM, is associated with disruption of gut homeostasis and gut barrier disturbances and has recently been revealed as a key susceptibility factor for neurological disorders [6]. Indeed, preclinical analysis of mice has recently suggested a potential link between changes in GM composition and AD [8]. A summary of all main findings of GM alternations from 16S rRNA sequencing in different pre-clinical AD models and the detrimental and beneficial effects of these organisms are shown in Tables **1** and **2** respectively. Zhang *et al.* found an increase in the pattern of *Firmicutes* and *Proteobacteria* in both aging wild type (WT) and AD littermate mice, whereas *Verrucomicrobia* was dramatically increased in old AD mice [9]. Another profound experiment conducted on mice suggested that increased Aβ peptide exposure is an early trigger of a pathway that leads to aggravated AD pathology, which in turn promotes poor gut homeostasis, and individual OTUs associated with *Allobaculum*, *Prevotella*, *Anaeroplasma*, *Bacteroides*, *SMB53*, and *Turicibacter* significantly increased in 5xFAD mice as compared to WT littermates [10]. In addition, recent data has shown that the transmission of GM from AD-mice (3xTg or 5XFAD mice) by either cohousing or transplanting AD-mice GM into young mice (3xTg mice or WT mice) changes mice’ behavior [11, 12]. Indeed, an old microbiome transplanted in antibiotic treated young mice stimulates C/EBPβ/AEP signaling in the brains, decreases brain synapses, and accelerates the development of cognitive impairment in young recipient mice [12]. As a proof of concept of the important role of the gut microbiome in AD, Grabrucker *et al.* revealed how FMT in the treatment of Alzheimer’s disease induced cognitive deficits in hippocampal-neurogenesis dependent behaviors in rats [13]. Furthermore, a study on APP^swe^/PS1^ΔE9^ (PAP) found that alterations in the intestinal microbiota are linked to cognitive impairment and enhanced amyloid deposition in the brain through triggering the MAPK signaling pathway [14]. In another study, *Firmicutes* were increased in the 5xFAD model mice while *Bacteroidetes* were decreased in comparison to the WT littermates. Additionally, the *Clostridium leptum* group was significantly increased in the 5xFAD mice with human amyloid-β protein precursor (AβPP) expressed in the brain and gut at the age of nine weeks old [15]. Furthermore, the composition of GM showed differences related to sex in Tg mice, adding more complexity when analyzing the impact of gut microbiome in AD disease was analyzed. A decrease in the abundance of *Ruminococcaceae* was linked to cognitive deficits in Tg-female mice [16]. Another study on Tg-APP/PS1 mice found a dramatic alteration in specific microbiota at all taxonomy levels, such as increasing *Firmicutes* along with decreasing *Proteobacteria* and *Bacteroidetes* at the phylum level by metagenome analysis using bacteria-derived membrane vesicles in blood [17]. Another study on the SAMP8 mice model indicated that SAMP8 mice had a notably different GM composition compared to SAMR1, and the pseudo germ-free mice receiving fecal transplants from SAMR1 mice showed improvements in behavior and GM diversity [18]. With the advancement in medical technology and profiling techniques like 16SRNA sequencing, Metagenomics and other multiomics approaches have revealed the various GM genera that might play an important role in AD. Changes in these bacterial phyla are linked with increased gut permeability which leads to leaky gut and gut dysbiosis. Additionally, they are also responsible for increasing Aβ accumulation in the brain and cognitive deficits. For instance, *Bifidobacterium Lactis Probio-M8* reduced Aβ plaque burden in the whole brain and protected against GM dysbiosis resulting in the alleviation of cognitive impairment in the APP/PS1 mice [19]. Recent evidence hypothesized that AD might be initiated in the gut by enteric neuron disfunction, which could lead to migration of Aβ to the brain and later induce brain pathology later. Aβ_1-42_ oligomers were injected into the stomach wall of 2-month-old healthy ICR mice. Then, the amyloid beta oligomer translocated from the colon to the brain over the course of a year. It was found that, Aβ oligomer translocation from the gut to the brain may play a key role in the development of AD and neuroinflammation [20].

It has become abundantly clear that the GM plays a complex role not only in regulating local gut homeostasis but also abroad in other tissues such as the brain. The intricate network of the so-call gut-brain axis is not only connected *via* GM. It also involves their metabolites, the enteric nervous system (ENS), the vagus nerve, and immune cells. GM influences peripheral immune cells such as T cells, IL-17 producing T cells, Ly6C^+^ monocytes, and Treg cells to maintain gut homeostasis or to increase a pro-inflammatory immune response during gut dysbiosis [21]. The altered GM composition promotes an aberrant rise of amino acids, particularly phenylalanine and isoleucine, as AD progresses. These amino acids encourage peripheral Th1 cells to infiltrate the brain *via* blood circulation. Local interaction between invading peripheral Th1 cells and M1 microglial cells in the brain results in severe neuroinflammation and cognitive impairment [22]. Shukla *et al.* reported that an unfavorable GM is linked to abnormally high gut NLRP3 (NOD-, LRR- and pyrin domain-containing protein 3) expression, which leads to peripheral inflammasome activation, which exacerbates AD-related neuroinflammation in 5XFAD mice [23]. In addition, studies have demonstrated that some of the metabolites produced by the GM known as short-chain fatty acids (SCFAs) are critical mediators in brain disease [24], suggesting that AD might not be entirely an Aβ-driven brain disease.

Genetic and epigenetic alterations, in addition to Aβ deposition and recruitment of pro-inflammatory immune cells and cytokines, are substantial risk factors for Alzheimer's disease. APOE genotype is associated with decreased butyrate-producing gut microbiome profiles and SCFAs, which affects the host metabolism and ultimately contributes to AD pathology [25]. Growing data suggests that epigenetic mechanisms (such as DNA methylation) play a role in Alzheimer's disease. Changes in DNA methylation, which involve the addition or removal of methyl groups at CpG dinucleotides, can affect gene expression, and it has been demonstrated that dynamic changes in DNA methylation occur across tissues in humans, nonhuman primates, and mice during ageing. Increased hippocampus DNA methylation is linked with the gut microbiome [26]. Through epigenetic alterations in AD-susceptibility genes in brain tissue, particularly gut microbiome genera that belongs to *Lachnospiraceae*, *Ruminococcaceae* and *Muribaculaceae family* are significantly associated with AD-relevant behavioral and cognitive function in APP mice [26].

Furthermore, male and female mice show differences in the composition of the GM throughout the progression of the AD brain pathology. With females having a greater burden of Aβ deposition due to the fact that the composition of microbiota within male and female murine models is subject to modulation by sex-specific hormonal profiles. Furthermore, the phenotypic expression of Alzheimer's disease pathology exhibits sexual dimorphism [27, 28]. At each age, the ratio of *Firmicutes/Bacteroidetes* remained lower in WT female mice with AD, suggesting that females more often show an increase in *Bacteroidetes* and a reduction in *Firmicutes* as opposed to male AD mice [29]. Gram-negative bacteria families such as *Bacteroidaceae* are both increased and reduced in male mice in relation to AD [30, 31], but more often increased in females [32]. Several studies showed that families comprising butyrate-producers like *Lachnospiraceae* [33] increased in male mice, whereas studies involving females or both genders showed a reduction [34].

One of the current key preclinical experiments that can be performed to demonstrate the effect of the gut microbiome on disease and explore some mechanistic questions around the topic, is by performing fecal microbiota transplantation (FMT) or cecal microbiome transplantation from healthy mice into diseased mice by increasing overall diversity and restoring the function of GM. In fact, FMT from healthy mice into ADLP^APT^ recipient animals, has been shown to restore diversity of gut microbiome, corrected the aberrant expression of intestinal macrophage activity-related genes and the rise of circulating blood inflammatory monocytes, as well as reduce amyloid plaque and neurofibrillary tangles (NFTs) production, glial responses, and cognitive impairment [35].

Although, the FMT approach has proven an important role of gut microbiome in AD, the mechanisms of gut microbiome-brain interactions in AD are still unknown and more research needs to be done, particularly on humans, considering the fact that the preclinical AD mice models currently available can only mimic the AD brain pathology that comes with AD. The analysis of gut microbial metabolites such as SCFAs is becoming one of the novel areas to explore in pre-clinical AD mice models. A few initial studies have suggested that microbiome derived SCFAs promotes Ab deposition in the brain along with pronounce alterations in the microglia. Conversely, germ free (GF) mice exhibit a significant reduction in AD brain pathology [36].

## 3 AD PATIENTS AND GUT MICROBIOME COMPOSITION

More evidence has arisen that GM is involved in promoting AD onset and progression in humans. The GM is linked to inflammatory and metabolic pathways in a variety of ways. Dysbiosis influences the synthesis of signaling proteins, which affects metabolic pathways associated with the progression of AD. Pro-inflammatory GM can contribute to the activation of innate immune cells such as macrophages and microglia thus resulting in the release of proinflammatory cytokines, such as interleukin-1 beta (IL-1β) and tumor necrosis factor-alpha (TNF-α), which leads to increased permeability of gastrointestinal tract and potentially exacerbates neuroinflammation in AD [37]. Conversely, anti-inflammatory microbiota might have a regulatory effect on the immune system. Beneficial bacteria, such as those producing SCFAs can modulate immune responses by promoting regulatory T cells and reducing the production of proinflammatory cytokines [38]. This modulation might have a protective effect against neuroinflammation and AD pathology by decreasing excessive immune activation. The normal intestinal barrier is comprised of tight junctions between epithelial cells, mucus, bicarbonate, and anti-microbial peptides secreted from Paneth cells. In AD patients, dysregulation of intestinal microbiome elevates production of cytokines and inflammatory mediators. These compounds further increase the permeability of the intestinal mucosa and the permeability of the brain blood barrier (BBB). Also, these cytokines make their way through circulation or vagus nerves to the brain, increasing the neuroinflammatory responses, and promoting neurodegeneration in CNS. P-glycoprotein (P-gp), a multidrug transporter, is highly expressed on the surface of intestinal epithelial cells and serves a crucial role in suppressing overactive inflammation to maintain intestinal homeostasis. Szabady *et al* found that elders with AD showed lower P-gp expression levels, which indicates gastrointestinal inflammation in AD [39]. These findings support the idea that changes in GM composition and related intestinal inflammation can correlate with AD severity and disease progression.

**Fig. (1):**
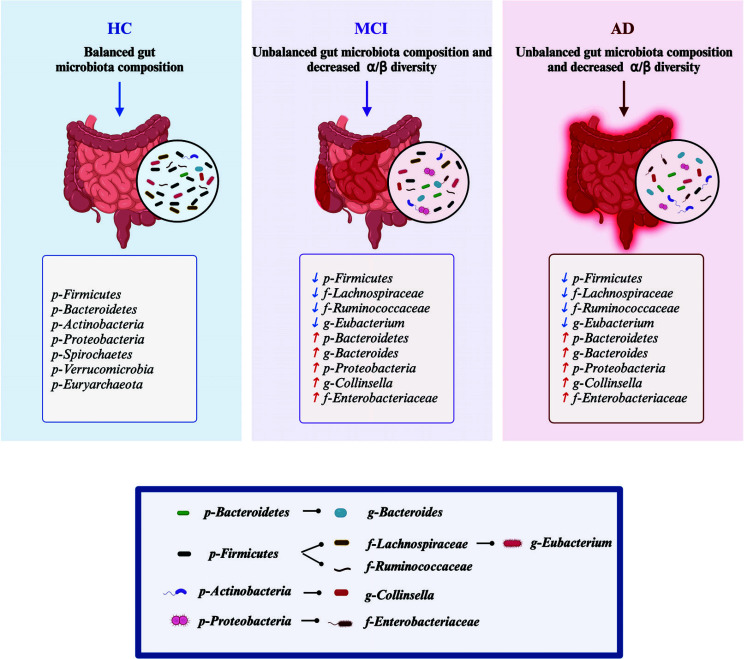
The model of the dynamic alterations of gut microbiota in the HC, AD and MCI patients. With the progression of AD, the results showed that *Bacteroidetes*, *Proteobacteria* and *Actinobacteria* at the phylum level, *Enterobacteriaceae* at the family level and *Bacteroides* at the genus level had an increasing trend. *Firmicutes* at the phylum level, *Lachnospiraceae* and *Ruminococcaceae* at family level, and *Eubacterium* at genus level had a decreasing trend. HC: healthy control; MCI: mild cognitive impairment; AD: Alzheimer's disease; *p-Bacteroidetes*: phylum *Bacteroidetes*; *g-Bacteroides*: genus *Bacteroides*; *p-Firmicutes*: phylum *Firmicutes*; *f-Lachnospiraceae*: family *Lachnospiraceae*; *f-Ruminococcaceae*: family *Ruminococcaceae*; *g-Eubacterium*: genus *Eubacterium*; *p-Actinobacteria*: phylum *Actinobacteria*; *g-Collinsella*: genus *Collinsella*; *p-Proteobacteria*, phylum *Proteobacteria*; *f-Enterobacteriaceae*: family *Enterobacteriaceae*.

Recently, many studies have reported that AD and mild cognitive impairment (MCI) in patients are often characterized by decreases in intestinal microbial diversity, with progressive growth of pro-inflammatory bacteria (such as *Verrucomicrobia*, *Escherichia/Shigella*, *Proteobacteria*, and *Pseudomonas aeruginosa*) and decreases in the abundance of anti-inflammatory bacteria (such as *Eubacterium hallii*, *Bacillus fragilis*, *Bacteroides fragilis*, *Eubacterium rectale*, *Faecalibacterium prausnitzii*, and *Bifidobacterium*) compared with controls. The GM composition in AD patients has been reported (Table **3**). Ling *et al.* [40] performed fecal microbiota 16S rRNA sequencing on 100 AD patients and sex matched 71- year- old healthy controls, a significant decrease in *Firmicutes* was observed accompanied by an increase in *Actinobacteria* and *Verrucomicrobia* at bacterial phylum level in AD patients compared to healthy controls. Interestingly, the abundance of butyrate-producing genera such as *Faecalibacterium* reduced significantly, while abundant lactate-producing genera, such as *Bifidobacterium*, increased significantly. Similar findings revealed lower relative abundances of butyrate-producing species and higher relative abundances of genera known to cause proinflammatory states in older people with AD compared to those with no dementia by metagenomic sequencing [41]. Moreover, 75 patients with MCI, the early stage of AD, also showed lower microbial diversity and higher abundance of *Proteobacteria* at the phylum level compared to 52 healthy participants from China [42]. Similarly, 16S rRNA sequencing was performed on 97 individuals (33 AD, 32 MCI and 32 controls), it revealed a pronounced difference in *Enterobacteriaceae* between MCI and AD patients, thus indicating that *Enterobacteriaceae* was associated with the presence and progression of AD [43]. Whereas significant decrease in *Firmicutes* and *Bifidobacteria*, and abundance of *Bacteroidetes* in the fecal samples of AD patients were found in another cohort study [44]. Moreover, Zhuang *et al.* compared 43 AD patients with age- and sex-matched controls and found enriched *Bacteroidetes* and decreased *Actinobacteria* at the phylum level with nearly same level of *Firmicutes* in the two groups [45]. Though, these studies did not mention which specific *Enterobacteriaceae* was involved in AD, other research reported *Escherichia coli* (*E. coli*), a *Enterobacteriaceae* member, considered as a pro-inflammatory bacteria due to their main endotoxin component LPS, to be enriched in perinuclear region and the hippocampus and neocortex of postmortem brains from AD patients [46, 47]. Moreover, some bacterial infections from *Enterobacteriaceae* family, such as *Salmonella* and *Shigella*, may contribute to the neurodegenerative process, which is associated with gut barrier dysfunction and increased intestinal permeability [48, 49]. In contrast to this evidence, a study identified 11 genera from the feces, but no difference in genera between AD and MCI was detected [50]. Another study found a significant decrease in *Proteobacteria* was found in AD patients compared with both MCI and healthy controls, while *Firmicutes*, *Bacteroidetes*, *Verrucomicrobia*, and *Fusobacteria* were not different among the three groups [51]. Furthermore, Cammann, D. *et al.* utilized a large genome-wide association study to analyze the genetic correlation between 119 GM genera and AD using polygenic risk score analyses. 20 of these 119 genera were initially identified as genetically associated with AD, and three genera (*Eubacterium fissicatena* as a protective factor, *Collinsella*, and *Veillonella* as a risk factor) were independently significant and notably, the *Collinsella* genera positively correlated with the *APOE* rs429358 risk allele, suggesting that proinflammatory gut microbiome could promote AD through interactions with *APOE* [52]. An illustrative model was used to depict the important GM differences among healthy controls and patients with AD and MCI (Fig. **1**). Moreover, the opposite findings of the GM composition in AD patients can be explained by the different geographical origin of the participants, since regional identity as well as local food and gender may strongly affect GM composition. However, no studies have yet elucidated whether there are gender-specific differences in GM in female and male AD patients. Some clinical studies only aimed to control for sex differences by comparing male and female AD patients. There is a need for targeted studies specifically comparing gender-specific GM in age- and sex matched healthy controls and, MCI and AD patients considering hormonal influences, immune responses and dietary habits of male and female AD cohorts.

Although most of the GM analysis of AD patients have been done by using 16S rRNA sequencing, a few small studies on AD patients have been able to confirm further findings by using Shotgun metagenomics. Laske *et al.* were able to discriminate amyloid-positive AD patients from healthy controls by combining taxonomic, functional intestinal microbiome data and clinical data using shotgun metagenomics, and showed that from the analyzed 18 genera 10 of them belonged to the phylum *Proteobacteria* [53]. Sohyun *et al.* revealed differentially abundant microbiota and metabolic pathways between 48 AD patients (38 dementia and 10 amnestic MCI) and 50 healthy controls by whole-genome shotgun metagenomic sequencing showing increased abundances of phyla *Firmicutes* and *Actinobacteria* and decreased abundances of *Bacteroides* and *Acidobacteria* in AD subjects [54]. One clinical study has suggested a correlation between amyloid load and blood SCFA concentration [55] suggesting the impact of GM-derived metabolites in AD. Supporting a more recent study revealed the associations between gut microbiome-dependent metabolites and neurodegenerative diseases, and found that glutamine was related to lower risk of AD [56]. Trimethylamine N-oxide (TMAO) is a microbial-dependent metabolite, which can enter the systemic circulation and has been found to correlate with the gut microbial composition. Nicholas *et al* showed that cerebrospinal fluid (CSF) TMAO is higher in individuals with MCI and AD dementia compared to healthy controls and elevated CSF TMAO is associated with biomarkers of AD pathology and neuronal degeneration [57]. Besides, GM can directly and indirectly regulate two major tryptophan metabolism pathways, the serotonin pathway and the kynurenine pathway (KP). Accumulating evidence indicates the involvement of KP in AD progression and inflammatory responses. In comparison to healthy controls, AD patients have higher plasma concentrations of the cytotoxic quinolinic acid and reduced concentrations of tryptophan and neuroprotective kynurenic acid [58]. Importantly, indole-3-pyruvic acid, a metabolite from tryptophan, was identified as a signature for discrimination and prediction of AD. It was found that the edge of senile plaques in the hippocampus of post-mortem AD brain tissue of AD patient has the greatest amounts of indoleamine 2, 3-dioxygenase (IDO) [59]. Kaddurah-Daouk *et al* also revealed that changes in tryptophan, as well as methionine, tyrosine, and purine metabolism occurred in CSF of MCI and AD patients, suggesting it might be a risk factor for cognitive decline [60]. Interestingly, Ferreiro *et al* showed GM composition correlated with Aβ and tau pathological biomarkers but not with biomarkers of neurodegeneration by metagenomic sequencing, suggesting that the GM may change early in AD progression [61]. Further understanding of possible mechanisms to explain the role of GM in AD subjects is scarce and is needed to better understand how changes in gut microbiome can impact other components of the gut-brain axis in AD patients, including intestinal epithelial layer, immune cells and neuroenteric system.

## 4 ASSOCIATION BETWEEN ANTIBIOTICS AND AD

In the presence of intestinal dysbiosis, the GM-derived metabolites and peripheral immune cells can cross the BBB and activate glial cells and the neuroinflammatory pathways in the brain. These interaction can lead to the Aβ burden and tau accumulation, triggering neurodegeneration [62]. Therefore, another important hypothesis is that the involvement of the antibiotics may have complicated effects on AD by altering GM composition. Several studies have demonstrated that different antibiotic treatments result in short- and/or long-term changes in the GM in both humans and animals. Moreover, antibiotic-induced gut dysbiosis is linked to changes in behavior and brain chemistry [63]. One study showed that administering a long-term broad-spectrum combinatorial antibiotic to APP/PS1 transgenic mice can elevate the levels of neuroinflammation and cytokines and reduce plaque-localized gliosis and alter microglial morphology, thereby exacerbating the progression of AD [64]. Some antibiotics, like streptozotocin and ampicillin, can disrupt the balance of gut bacteria and cause neurocognitive disorders. The rats showed increased levels of serum corticosterone, and impaired spatial memory after being given ampicillin [65]. Surprisingly, the use of probiotics (specifically *Lactobacillus fermentum strain NS9*) can reverse the physical and mental irregularities caused by ampicillin in rats. Besides, Streptozotocin has been utilized to trigger sporadic forms of AD in animal models, impacting their learning and memory abilities [66]. Another animal research found that antibiotic-treatment depleted and restructured GM composition of cecal contents from weaning onwards leading to decreased anxiety, cognitive impairments, and changes in the dynamics of the tryptophan metabolic pathway [67]. In humans, certain antibiotics such as cefepime can penetrate the blood-brain barrier, leading to symptoms such as altered mental status, decreased consciousness, myoclonus, and confusion [68]. One population-based prospective cohort study suggested that middle-aged women who take antibiotics continuously for two months may experience cognitive decline afterward [69]. This indicates that antibiotics may not be beneficial for all AD patients, especially those without a clear history of microbial infection. However, antibiotics may also have positive impacts on AD, such as reducing neuroinflammation caused by dysbiosis. These effects include neuroprotection, anti-inflammatory, anti-tau, anti-amyloid, and cholinergic effects, regardless of whether they are directly caused by antibiotics. For instance, administering rifampicin orally to three different mouse models of AD and tauopathy decreased the accumulation of Aβ oligomers and tau oligomers, and improved the memory of the mice [70]. Preliminary research using the TgCRND8 transgenic mouse model revealed that administering erythromycin in drinking water for 3 months resulted in a 54% reduction in Aβ 1-42 levels in the cortex compared to controls [71]. Also, research indicates that minocycline can reduce the neuroinflammatory response of microglia induced by Aβ oligomers by preventing their activation into a pro-inflammatory state [72]. Moreover, the triple antibiotic therapy (omeprazole, clarithromycin, and amoxicillin) to eliminate Helicobacter pylori has shown promising outcomes in improving cognitive and functional status parameters in AD patients [73]. Furthermore, a study conducted on AD patients showed improvement in the cognitive subscale of the Standardized AD Assessment Scale when patients received treatment with both doxycycline and rifampicin over a period of 6 months [74]. At present, there is lack of scientific evidence for the use of antibiotics as therapeutic agents for AD. Dose and regimen are issues that cannot be ignored in the use of antibiotics, and the action of antibiotics in AD depends on the type of antibiotic and on the specific role of the microbiome in AD pathogenesis.

## 5 EVIDENCE OF DIETARY EFFECTS ON AD PATIENTS

Adding more complexity into the impact of GM in AD. Diet is considered as a modifiable environmental factor with a detectable influence on microbiota composition, which has been thought to strongly affect AD pathogenesis [75]. Varesi *et al.* discussed the therapeutic potential of improved dietary regimens could be effective alternatives for preventing and delaying the etiology and progression of Alzheimer's disease [76]. Therefore, we present some of the most promising dietary therapies, which particular focus on Mediterranean diet, dietary approaches to stop hypertension, Mediterranean-DASH intervention for neurodegenerative delay, and ketogenic diet.

### 5.1 Mediterranean Diet

The Mediterranean diet (MedDiet) is considered a healthy dietary plan, which features a high intake of vegetables, fruit, whole grains, nuts, and olive oil, moderate consumption of fish and poultry and limited consumption of red meat and sweets. Recent evidence has shown that higher adherence to the MedDiet was associated with less cognitive decline and a lower risk of AD [77]. Sun *et al.*, showed how higher adherence to a MedDiet was related to a 17% reduced risk of developing MCI and a 40% reduced risk of AD [78]. A meta-analysis involving 15 cohort studies with 41,492 participants and 2 randomized control trials (RCTs) also revealed strong evidence of the beneficial effect of the MeDiet on older adults' global cognition [79]. In addition, several studies from multiple countries and regions concluded that a higher adherence to the MedDiet was strongly related to better cognitive function, slower onset of cognitive decline, and reduced risk of developing MCI, AD, and dementia [80-83]. However, differential results from other two RCTs did not show any significant association. Knight *et al.* followed up with 137 men and women and found that the MedDiet group did not perform significantly better than the control group in terms of cognitive function [84]. Another earlier RCT indicated that the MeDiet was not significantly associated with motor speed, memory, or choice reaction time compared with the control diet [85], confirming the importance of more research on mechanisms of diet at influencing how we age. Generally, most evidence on MeDiet showed promising ability to hinder cognitive impairment. However, he role of DASH diet in AD prevention and therapy is not fully understood. A recent study showed that adherence to the MedDiet selectively increased the abundance of SCFA-producing and anti-inflammatory bacteria, which were associated to improved cognitive function in older people. Besides metabolite profiles indicated that microbiome change was related with increased SCFAs and branched fatty acids and lower production of secondary bile acids, p-cresols, ethanol and carbon dioxide as well as reduced inflammatory markers, including C-reactive protein and interleukin-17 (IL-17) [86]. Moreover, some studies also indicated that the MedDiet selectively promoted the growth of *Prevotella* and *Bifidobacteria*, as well as some fiber-degrading bacteria, such as *Faecalibacterium prausnitzii*, *Eubacterium eligens* and *Bacteroides cellulosilyticus* and reduced the levels of proinflammatory bacteria such as *Ruminococcus gnavus*, leading to higher levels of SCFAs and lower levels of metabolic endotoxemia in humans [87, 88]. Furthermore, SCFAs are capable of inhibiting Aβ oligomerization thus suppressing the transformation of monomeric Aβ_40_ and Aβ_42_ into Aβ fibrils, thereby reducing neurotoxicity in the brain [89].

### 5.2 (DASH) Diet: Dietary Approaches to Stop Hypertension

DASH diet is an accepted dietary pattern applied to prevent and treat hypertension and improve cardiovascular disease [90]. The DASH diet also promotes a high intake of plant-based foods. However, the DASH diet emphasizes minimal dietary sodium, sweetened beverages, and red meats and does not encourage alcohol [91]. So far, the reported effects of the DASH diet on neurocognition have not been consistent. A prospective study found that high DASH adherence was associated with consistently higher levels of cognitive function in 3831 elders [92]. However, a RCT demonstrated that the DASH diet alone has no significant benefit in adults with cognitive impairment [93]. So far, the role of DASH diet in GM composition and AD prevention and therapy is not fully determined.

### 5.3 Mediterranean-DASH Intervention for Neurodegenerative Delay (MIND) Diet

The MIND diet is a combination of the MedDiet and the DASH diet and aims to reduce dementia and the decline [94]. Many observational research and clinical trials indicate that the MIND diet can improve brain cognitive function and prevent dementia. A recent review included 13 studies (9 cohort, 3 cross-sectional, and 1 RCT) and revealed that adherence to the MIND diet may possibly be associated with an improved cognitive function in older adults. Furthermore, MIND diet may be superior to other plant-rich diets for improving cognition [95]. One of the large sample (5907 elders) cross-sectional studies found that the MIND was associated with better cognitive function and reduced the risk of developing AD by 53% [96]. Moreover, a longitudinal cohort study showed that the MIND diet reduced odds of 12-year cognitive impairment [97]. Despite these promising results, the mechanism linking MIND diet and cognitive performance remains unclear, which may be related to the reduced levels of oxidative stress and inflammation and decreased Aβ protein in the brain [95].

### 5.4 Ketogenic Diet

The ketogenic diet (KD) is an alimentary regimen characterized by high fat and very low carbohydrate intake sufficient to enhance ketone production. Recently, KD has been found to be beneficial to people with mild cognitive impairment or AD. A recent review summarized 14 of 15 studies and indicated that administration of ketogenic diets or ketone bodies in patients with MCI, mild-to-moderate AD or AD induces significant improvement in cognitive function [98]. For instance, a recent RCT recruited gave 83 MCI patients with intervention of ketogenic drink and followed for 6 months, and they found ketogenic drink improved cognitive outcomes in MCI patients [99]. Besides, some studies concluded that KD had positive effects on verbal memory visual attention and processing speed both in non-demented elderly and AD patients [100, 101]. The potential mechanisms responsible for the beneficial effects of the KD on cognitive function also involve the change of GM. A recent RCT study found that modified Mediterranean-KD can modulate the gut microbiome and metabolites in association with improved AD biomarkers in CSF. The favorable microbiota profiles including *Enterobacteriaceae, A. muciniphila*, *Slackia*, *Christensenellaceae* and *Erysipelotriaceae* were enriched, and fecal propionate and butyrate levels increased, which are negatively associated with Aβ42 brain pathology. In contrast, the reduction of *Proteobacteria* was positively associated with Aβ42/Aβ40 in subjects with mild cognitive impairment [102].

In conclusion, MedDiet, MIND, DASH and KD show positive effects in preventing or slowing age-related cognitive decline and alleviating the occurrence of AD based on clinical evidence despite some limitations. Further studies need to determine which dietary pattern has the best efficacy and identify when to change the pattern so that it may have the greatest impact on AD. Based on the effects of dietary patterns that may involve the roles of GM, further research is essential to explore whether dietary interventions for the prevention of AD would be mediated by GM.

## 6 GUT MICROBIOME-TARGETED THERAPIES IN AD

### 6.1 Fecal Microbiota Transplantation

**Fig. (2):**
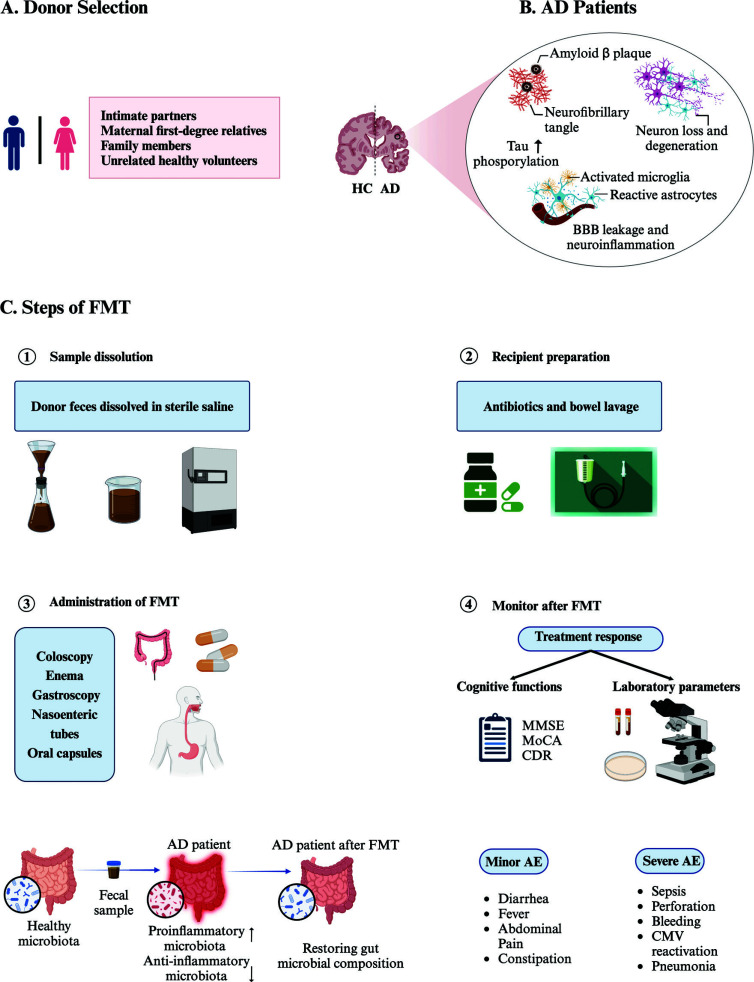
Schematic representation of the standard FMT protocol in humans. (**A**) The standard of donor selection for FMT. It is recommended that the donors for FMT should be selected from intimate partners, maternal first-degree relatives, family members, or unrelated healthy volunteers. (**B**) Neurodegenerative pathology in AD. AD is a neurodegenerative disease characterized by progressive memory loss, cognitive impairment, and changes in personality. Pathology includes the presence of misfolded amyloid-beta (Aβ) plaques, deposition of hyperphosphorylated tau as neurofibrillary tangles, loss of neurons and activated microglia and astrocytes. (**C**) How to administrate FMT. There are several steps to administrate FMT including fecal material preparation, recipient preparation, fecal delivery and monitoring the treatment of response and adverse events after FMT. The fecal sample of AD patients showed increased proinflammatory microbiota and decreased anti-inflammatory microbiota, which can be restored to be normal gut microbial composition after FMT. **Abbreviations**: FMT: fecal microbiota transplantation; AD: Alzheimer's disease; HC: healthy control; BBB: blood-brain barrier; MMSE: Mini-Mental State Examination; MoCA: Montreal Cognitive Assessment; CDR: Clinical Dementia Rating; AE: adverse events; CMV: cytomegalovirus.

FMT involves transferring a suspension of fecal material from a healthy donor into the intestinal tract of a recipient (by colonoscopy, nasogastric tube, or oral tablets), with the goal of directly modifying or “restoring” the GM composition to avoid dysbiosis (Fig. **2**). Potential donors for FMT can be selected from intimate partners, family members, or unrelated volunteers, and need to undergo the selection process. The selection process usually includes collection of medical history, blood testing and stool testing to check donors for any potentially transmittable disease. Both fresh feces and frozen feces can be used for FMT. Although the appropriate weight of feces for FMT has not been defined, 50 to 60 g of stool is recommended for each treatment. Moreover, fecal materials need to be diluted with sterile saline solution (0.9%) with three to five times larger volume of solvent (*e.g*., 30 g of feces to be diluted in 150 mL of saline). Then, fecal material should be suspended in saline using a blender or manual effort and sieved to avoid the clogging of infusion syringes and tubes. The fresh fecal material can be used within 6 hours after preparation or should be clearly labeled and stored at -80°C. On the day of the infusion, it should be thawed in a warm water bath at 37°C and infused within 4 hours of thawing [103]. The traditional delivery of FMT is by colonoscopy or *via* upper GI tract, such as gastroscope, or through nasogastric, nasojejunal or gastrostomy tube. Recipients should be prepared with bowel lavage when FMT is performed by upper route or by colonoscopy. Capsule delivery is the most recent modality of FMT, which seems to be favorable for those with colonoscopy or gastroscope contraindication. After FMT, recipients should be monitored for the occurrence of possible acute complication. FMT is generally regarded as a secure and effective therapy approach with few adverse effects. Most common short term adverse events after FMT, such as diarrhea, constipation, abdominal pain, bloating, and transient low-grade fevers, are likely to resolve within few days to weeks. Uncommon but serious side effects include pneumonia, sepsis, intestinal pathogen transmission, perforation, hemorrhage, cytomegalovirus (CMV) reactivation, and high-grade fever are associated with endoscopy and sedation [104]. Moreover, it is essential to follow up on the treatment response of AD patients. Treatment response implies clinical improvement, such as improvement of cognitive function testing (Mini-Mental State Examination, Montreal Cognitive Assessment, and Clinical Dementia Rating assessment), as well as amelioration of other parameters of disease severity such as laboratory parameters and radiological examination [105]. Nevertheless, there is currently no biomarker easy to track in peripheral blood to follow up with patients and predict outcomes.

A carefully designed FMT study can provide functional evidence that GM can be implicated in CNS changes in AD disease. Only two case studies with encouraging findings have been undertaken for AD patients. Hazan *et al.* [106]. found that FMT from 85-year-old woman (recipient’s wife) improved AD symptoms (cognitive function, memory, and mood) in an 82-year-old male. A second case study with a 90-year-old woman with Alzheimer's disease and severe C. difficile infection who got FMT from 27-year-old healthy male revealed improvements in cognitive function, microbiota diversity, and SCFAs production [107]. Nevertheless, the studies are scarce, and large multicenter control/cases cohort studies that account carefully for variables such as diet, sex, age, ethnicity, geographical location and AD clinical stage, are still needed.

### 6.2 Prebiotics

Prebiotics, on the other hand, are described as nutrients that have been specifically fermented to cause specific changes in the composition and/or activity of the GM, bringing health advantages to the host [76, 108]. Several studies have suggested that prebiotics can promote the growth of beneficial bacteria such as *bifidobacteria* and *lactobacillus*, improving gut dysbiosis and its inflammatory-associated state [109]. Preclinical and clinical data showed that prebiotics also play a positive role in the CNS by through modulating neuroinflammation, which alleviate cognitive deficits, depression, and anxiety [110]. Recently, some studies have revealed the promising results of the use of prebiotics in the prevention and treatment of AD. A longitudinal study that included 1,837 elderly (≥ 65 years) participant aimed to investigate the association between dietary prebiotic intake and risk for AD, showed that fructan intake was significantly associated with reduced AD risk in Hispanics [111]. Ahmed *et al.* conducted a RCT to investigate the impact of daily consumption of MSPrebiotic (a digestion resistant starch derived from potatoes: 20% amylose and 80% amylopectine) on gut microbiome composition. The study recruited 84 healthy elderly people, aged ≥ 70 years, which found that the prebiotics produced an increased abundance of endogenous *bifidobacteria* and prevented dysbiosis of gut *Proteobacteria* observed at baseline [112]. Another RCT study assessed the effect of galacto-oligosaccharides (GOS) in 40 healthy elderly volunteers, aged 65-80 years. The authors found positive effects on both the microbiota composition and the immune response, including significant increases in phagocytosis, NK cell activity, and the production of IL-10 and significant reduction in proinflammatory cytokines [113]. Walton *et al.* also conducted RCT to explore the role of GOS on the intestinal microbiome composition in elderly people, showing increased abundance of *bifidobacteria* and increased butyrate production, which may constitute beneficial modulation of the GM in a maturing population [114]. Although, the current data suggests a possible therapeutic role of prebiotics in modulating GM in AD, there is currently a shortage of evidence to conclude, and more human clinical trials are needed before drawing any conclusion.

### 6.3 Probiotics

Probiotics are living bacteria that, when administered in sufficient quantities, promote health benefits. Probiotics have been found to regulate intestinal ecosystem homeostasis, modulate intestinal epithelial functions by maintaining the epithelial barrier, supporte cell survival, generate SCFAs, and suppressing the generation of pro-inflammatory cytokines in human and animal models [115].

Probiotics also play important role in the gut-brain axis by modulating the bidirectional communication between the brain and gut, through the modulation of neurotransmitters and proteins, including gamma-aminobutyric acid, serotonin, glutamate, and the brain-derived neurotrophic factor, which play important roles in the functionality of our CNS, mood, cognitive functions, learning and memory processes [76, 116].

However, due to the lack of evidence of the modulation of probiotics in patients with AD, impact of probiotics on the onset symptoms and progression of AD remains unclear. Akbari *et al* [117] conducted a clinical trial to assess the probiotics in AD patients. AD patients received 200 ml/day of milk enriched with *Lactobacillus acidophilus*, *Lactobacillus casei*, *Bifidobacterium bifidum* and *Lactobacillus fermentum* (2 × 10^9^ CFU/g each) for 12 weeks. The results showed that probiotic treatment improved MMSE test, and counteracted oxidative stress, improved insulin resistance and pancreatic β-cell secretion and decreased serum triglycerides compared to controls. Similarly, Agahi *et al.* [118] provided 25 AD patients with probiotics in the dosage of 3 × 10^9^ CFU daily for 12 weeks. In the 12^th^ week, increased Test Your Memory (TYM) score, cognitive function, and serum glutathione (GSH), and lower levels of 8-OHdG in the serum of AD patients were observed. However, there was no significant difference in overall antioxidant capacity. Leblhuber *et al.* [119] administrated 20 AD patients with probiotic supplementation of *Lactobacillus* and *Bifidobacterium* for 4 weeks. The increase of kynurenine, nitrite, and neopterin and RNA content of *Faecalibacterium prausnitzii* in the fecal matter of AD patient and a reduction in zonulin concentration was observed in the study. Tamtaji *et al.* [120] conducted a randomised clinical trial, where 79 patients were randomly assigned to receive either selenium (200 μg/day) plus probiotic containing *Lactobacillus acidophilus*, *Bifidobacterium bifidum*, and *Bifidobacterium longum* (2 × 10^9^ CFU/day each) (n = 27), selenium (200 μg/day) (n = 26) or placebo (n = 26) for 12 weeks. The results showed improved MMSE score, increased GSH and antioxidant, and reduced high-sensitivity C-reactive protein (hs-CRP) and serum triglyceride. Interestingly, a positive correlation between probiotic supplements and with antioxidant capacity was found but contradicted both the findings from Akbari and Agahi. Though these exciting results suggest the positive effect of probiotic supplements on alleviating AD progression, the mechanisms of probiotics remain largely unknown, and several gaps and inconsistences remain. Further investigation with large-scale sample is needed to prove the effect of probiotic on AD.

## CONCLUSION

Altogether, the current evidence suggests that the characterization of GM gut microbiome composition may serve as a tool for early screening, diagnosis of prognosis in AD. However, the field is nascent and still controversial. Further studies aimed at integrating the GM data with genomic and metabolomic datasets in a larger clinical cohort of sex-, and age-matched controls, will be important to develop novel strategies focused on targeting the GM as a novel therapeutic strategy for AD. Larger cohort studies on humans will be required to fully understand how the gut microbiome can impact AD to later develop specific therapeutic strategies in AD.

## Figures and Tables

**Table 1:** Major findings of gut microbiota composition from 16S rRNA sequencing in pre-clinical models. Description of the most important findings of the gut microbiome composition found in several AD mice model at different ages.

**Mouse Model**	**Age of Mice**	**Main Findings**	**References**
APP^swe^/PS1^ΔE9^ (PAP)	8-12 months old	When 8-12-month-old AD mice were compared to age-matched wild type controls, the number of *Verrucomicrobia* and *Proteobacteria* was drastically increased, while *Ruminococcus* and *Butyricicoccus* were significantly decreased. *B. pullicaecorum*, a butyrate generator with probiotic potential, was found to be less abundant in AD mice. Butanoate/pyruvate metabolism was expected to be changed in AD mice by the functional analysis.	[9]
5XFAD	2 months old	Though community composition of the gut microbiome at the phylum level was highly similar in 5xFAD mice as compared to wild type littermates. At the genus level, *Allobaculum*, *Prevotella*, *Anaeroplasma*, *Bacteroides*, *SMB53*, and *Turicibacter* significantly increased in abundance in 5xFAD animals. At the species level, *Bacteroides acidifaciens*, *Lactobacillus reuteri*, and *Ruminococcus gnavus* enhanced in the 5xFAD group.	[10]
5XFAD	6 months old	Age-dependent dynamic modifications in the gut microbiota of 5xFAD mice, characterized by a reduction in microbial diversity, followed by a substantial depletion of anti-inflammatory *Firmicutes* and temporal enrichment of proinflammatory *Proteobacteria* such as *Helicobacter bilis*.	[12]
APP^swe^/PS1^ΔE9^ (PAP)	5 months old	In 5-month-old SPF PAP mice, microorganisms that generate short-chain fatty acids (SCFA), particularly butyrate, were depleted, such as *C. methylpentosum*, *A. butyrica*, *L. asaccharolyticus*, *K. alysoides, F.butyricus*, and *A. finegoldii*. *B. acidofaciens* levels were higher in the PAP mice. Furthermore, the concentrations of *H. ganmani* and *H. bilis*, which are linked to inflammation and cell death, also increased.	[14]
5XFAD	2 months old	Increased *Firmicutes/Bacteroidetes* ratio in 5X FAD mice. At nine weeks of age, the *Clostridium leptum* group was considerably elevated in the 5xFAD mice.	[15]
APP/PS1	6 months old	Gender differences have a distinct impact on the microbiome composition in AD pathogenesis. Gut dysbiosis in Tg-male mice was more severe than in Tg-female animals. In Tg-female mice, a decrease in *Ruminococcaceae* abundance was linked to cognitive impairments.	[16]
APP/PS1	8 months old	In Tg-APP/PS1 mice, the levels of *p_Firmicutes* increased whereas the levels of *p_Proteobacteria* and *p_Bacteroidetes* substantially reduced. Furthermore, the levels of *g_Aerococcus*, *g_Jeotgalicoccus*, *g Blautia*, *g_Pseudomonas*, and unclassified members of the *f_Clostridiale* and *f_Ruminococcaceae* increased, while the levels of *g_Lactobacillus*, unclassified members of the *f_S24-7*, and *g_Corynebacterium* decreased compared to control mice.	[17]
SAMP8	7 months old	The aberrant composition of the gut microbiota is linked to cognitive impairment in SAMP8 mice. The researchers discovered 27 microorganisms that differed between the faeces of SAMR1 and SAMP8 mice. In comparison to SAMR1 animals, the relative abundance of the species uncultured *Bacteroidales bacterium* was considerably higher in SAMP8 mice.	[18]

**Table 2:** Beneficial and detrimental effects of most relevant gut bacteria in Alzheimer’s disease. Description of the most relevant gut bacteria involved on pre-clinical studies on AD mice models.

**Bacteria Species**	**AD Disease** **Model in Mice**	**Positive Or** **Negative Effects**	**Type of Effects**	**References**
*Coriobacteriaceae* and *g_Clostridium*	APP^swe^/PS1^ΔE9^	Negative	Significantly increased Aβ plaques	[9]
*Helicobacter bilis*	5XFAD	Negative	The increase in these bacteria was positively correlated with the C/EBPB/AEP pathway in 6-month-old mice, leading to gut dysbiosis	[12]
*L. salivarius*	5XFAD	Positive	Attenuated gut leakage and inflammation and oxidative stress	[12]
*Bacteroidales_S24-7* group	SAMP8	Positive	Helped in maintaining a role in electron and oxidative stress to mediate host-microbe interactions	[18]
*Desulfovibrionales*, families *Christensenellaceae* and *Ruminococcaceae*, and genus *Desulfovibrio*	SAMP8 mice	Negative	Related to cognitive dysfunction	[18]
*Bifidobacterium Lactis* *Probio-M8*	APP/PS1 mice	Positive	Reduced Aβ plaque burden in the whole brain and protected against gut microbiota dysbiosis and alleviated cognitive impairment in the APP/PS1 mice	[19]
*Lactobacillus plantarum* and *Bifidobacterium infantis*	5XFAD	Positive	Enhanced the intestinal barrier by increasing the expression of the protein associated with tight junctions	[23]
*Bacteroides fragilis*	5XFAD	Negative	Disrupted adherent junctions by cleavage of cell adhesion molecules such as E-cadherin	[23]

**Table 3:** Gut microbiome composition in Alzheimer’s disease patients. Description of the most relevant papers available in the literature on characterization of gut microbiome in AD patients and the most significant increased or decreased bacteria phyla.

**Sample Size**	**Sequencing Technology**	**Increasing Bacterial Phylum**	**Decreasing Bacterial Phylum**	**References**
100 AD patients and 71 age- and sex- matched HC	16S rRNA Miseq sequencing of fecal microbiota	*Actinobacteria* and *Verrucomicrobia* in AD compared to HC	Bacterial diversity *Firmicutes* in AD compared to HC	[40]
75 MCI individuals and 52 heathy controls	16S rRNA gene sequencing and serum miRNA expression	*Proteobacteria* in MCI compared to HC	*Bacteroides* and *Firmicutes* in MCI compared to HC	[42]
33 AD patients, 32 MCI patients and 32 HC	16S rRNA MiSeq sequencing and phylogenetic investigation	*Proteobacteria* in AD compared with both HC and aMCI *Bacteroidetes* in aMCI compared with HC	Microbial diversity *Firmicutes* in AD compared with HC *Bacteroidetes* in AD compared with both MCI and HC	[43]
25 AD patients and 25 age- and sex-matched Control participants	16S rRNA gene sequencing	*Bacteroidetes* in AD compared to HC	Microbial diversity *Firmicutes* and *Actinobacteria* in AD compared to HC	[44]
43 AD patients and 43 age- and gender-matched cognitively normal controls	16S rRNA gene sequencing	*Actinobacteria* in AD compared to HC	*Bacteroidetes* and *Verrucomicrobi*a in AD compared to HC	[45]
30 AD patients, 30 MCI patients, 30 heathy controls	16S rRNA sequencing in the feces and blood	*Firmicutes* in AD compared to HC	*Bacteroidetes* in AD compared to HC	[50]
18 AD patients, 20 MCI patients, and 18 age-matched HC	16S rRNA gene sequencing	β-diversity in AD and MCI compared to HC	*Proteobacteria* in AD compared with both MCI and HC	[51]

## LIST OF ABBREVIATIONS

AD: Alzheimer’s Disease
CNS: Central Nervous System
CSF: Cerebrospinal Fluid
ENS: Enteric Nervous System
FMT: Fecal Microbiota Transplantation
GF: Germ Free
GM: Gut Microbiome
MCI: Mild Cognitive Impairment
NFTs: Neurofibrillary Tangles
RCTs: Randomized Control Trials
SCFAs: Short-chain Fatty Acids
